# Editorial: Inducing Plant Resistance Against Insects Using Exogenous Bioactive Chemicals: Key Advances and Future Perspectives

**DOI:** 10.3389/fpls.2022.890884

**Published:** 2022-04-18

**Authors:** Islam S. Sobhy, Yonggen Lou, Toby J. A. Bruce

**Affiliations:** ^1^School of Life Sciences, Keele University, Keele, United Kingdom; ^2^Department of Plant Protection, Faculty of Agriculture, Suez Canal University, Ismailia, Egypt; ^3^State Key Laboratory of Rice Biology and Ministry of Agriculture Key Lab of Molecular Biology of Crop Pathogens and Insects, Institute of Insect Science, Zhejiang University, Hangzhou, China

**Keywords:** induced plant defenses, tritrophic interactions, priming, insect herbivores, crop protection

Due to the constraints and hazards of using insecticides such as development of insect resistance, severe decline in availability of conventional pesticides and off-target effects on beneficial insects (Desneux et al., 2007), there is an urgent need to develop the underpinning science to protect crop harvests from insect pests in the face of rising demand for food (Savary et al., 2019). Given the recent advances in our understanding of plant-insect interactions, it is proposed that boosting the overall plant immunity could provide novel alternative control tactics. Constitutively increasing defense could have a negative trade-off with growth or yield (Huot et al., 2014) and therefore inducing resistance could be a more attractive prospect.

During their coevolution with insects, plants have evolved a complex arsenal of defense mechanisms against antagonistic herbivores while also attracting beneficial insects (Bruce, 2015). Some plant defenses are constitutive (i.e., always present in the plants) while others are induced after plant perception of stimuli associated with insect herbivory (Erb et al., 2012). Such inducible defenses against insect herbivores are regulated by the signaling of two major phytohormones, i.e. salicylic acid (SA) and jasmonic acid (JA) (Thaler et al., 2012). Typically, JA is associated plant defenses against chewing insects while SA induces resistance against piercing/sucking insects but there is considerable variation between different insect-plant systems (Erb et al., 2012).

The induction of both JA and SA pathways can also be achieved by applying bioactive chemicals that act as inducers of plant resistance against herbivores (Pickett and Poppy, 2001). An accumulated body of studies, from the last three decades, has explored inducing plant defense *via* the application of bioactive chemicals as a sustainable and ecologically sound approach to control insect pests in agriculture (Stout et al., 2002; Sobhy et al., 2014). Even though there has been mounting attention to the potential of using defense inducers, their exploitation in agricultural practice is still at its infant stage and therefore further development is required (Yassin et al., 2021). Given that, the objective of this Research Topic is to highlight recent progress, key advances, and future perspectives in manipulating plant defense against insect pests using bioactive chemicals. Fourteen articles were published in this Research Topic, and from these we only focus our editorial on ten papers covering the following research themes: (1) defense inducers impact on plant growth and yield; (2) dual effect of plant inducers on pathogen and insect pests; (3) genotypic variation in elicitor -induced defense; and (4) exploiting these chemicals as defense inducing or priming agents.

Little is still known about the possible negative trade-off effects of defense inducers on plant growth and yield under field conditions (Yassin et al., 2021). Four papers in the Research Topic address the impact of defense inducers on plant growth and yield. Bhavanam and Stout found that when seed treatment with JA and methyl jasmonate (MeJA) enhanced resistance of rice plants to rice water weevils but also reduced seedling emergence, plant height, and filled grain mass. Under field conditions, Yoshida et al. observed that whereas Japanese radish treatment with Prohydrojasmon (PDJ) induced direct and indirect defense against several insect pest species (e.g., aphids, leaf-mining fly larvae, vegetable weevils, and thrips), the biomass of both aboveground and belowground parts of PDJ treated plants was significantly lower than untreated plants. Similarly, Chen et al. provide another field evidence that MeJA significantly slowed down the growth of Conifer seedling relative to control. In contrast, Mouden et al. reported that strawberry growth was not affected by MeJA application as were fruit yield and quality whereas leaf damage by thrips was lower on treated plants. These four papers therefore support the idea that negative impact on plant overall growth may impede agricultural exploitation of plant defense inducers (Walters and Heil, 2007).

To prioritize one response over the other, plants under natural conditions exhibit a crosstalk between plant hormonal signaling pathways which may result in either synergistic or antagonistic effects (Spoel and Dong, 2008). Exogenous application of elicitors of pathogen resistance could lead to increased susceptibility to one or more attacking herbivores due to negative crosstalk between the SA and JA pathways (Sobhy et al., 2012; Thaler et al., 2012). Two papers in the Research Topic address SA and JA crosstalk following plant treatment with defense elicitors. Puentes et al. found that MeJA treatment not only increased resistance to the pine weevil *Hylobius abietis* but also enhanced the Norway spruce (*Picea abies*) resistance to the necrotrophic blue-stain fungus *Endoconidiophora polonica*. Using a trophic system comprising of cranberries, the phytoplasma that causes false blossom disease, and two herbivores—the blunt-nosed leafhopper (*Limotettix vaccinii*), the vector of false blossom disease, and the non-vector gypsy moth (*Lymantria dispar*), Rodriguez-Saona et al. evaluated the treatment effect of four commercial elicitors, including three that activate mainly SA-related plant defenses (Actigard, LifeGard, and Regalia) and one activator of JA-related defenses (Blush) on cranberries defense induction. They found that phytoplasma infection and elicitor treatment had positive effects on *L. vaccinii* and *L. dispar* performance in cranberries, likely *via* enhancement of plant nutrition and changes in phytohormone profiles, suggesting that the studied elicitors did not improve herbivore resistance or reduce phytoplasma infection in cranberries.

To advance practical use of defense activators, more consistent and repeatable responses to treatment are required. Plant responses to elicitor chemicals are variable due to differences in plant genotypes and environmental conditions they are deployed in, which is a main reason why it is challenging to use them widely in agriculture (Bruce, 2014). In this article collection, when Puentes et al. examined genotypic variation between nine clones of *P. abies* in MeJA-induced responses, they found that MeJA treatment increased resistance to *H. abietis* damage and *E. polonica* infection, but effects varied among clones depending on their constitutive resistance levels. In addition, using a model system comprising the generalist herbivore fall armyworm (FAW) *Spodoptera frugiperda* and three economically important plant species with differential ability to uptake silicon: tomato (non-Si accumulator), soybean, and maize (Si-accumulators), Acevedo et al. found that FAW herbivory and Si supply increased peroxidase (POX) activity and trichome density in tomato, and the concentration of phenolics in soybean suggesting variations in defense inducibility between plant species. The same pattern was also reported by Ali et al. who investigated the effect of treating a range of Brassica cultivars with the defense activators *cis*-Jasmone (CJ) on tritrophic interactions with *Myzus persicae* aphids and their parasitoid *Diaeretiella rapae*. They found that CJ treatment made plants less attractive to and less suitable for *M. persicae* but more attractive to *D. rapae* in certain brassica cultivars due to variation of emitted volatile profiles upon CJ treatment.

Defense priming allows plants to deploy induced defenses more rapidly and robustly when subsequently challenged by future insect attacks (Conrath et al., 2015) and can be triggered using certain compounds called priming agents (Sobhy et al., 2018). In this collection, two papers addressed this phenomenon under greenhouse and field conditions. Shiojiri et al. conducted a field experiment for 2 years in which rice seedlings were exposed to artificially damaged weed volatiles and then leaf damage was observed. They found that total number of damaged leaves in volatile-exposed plants was significantly lower but their grain weight per bunch was significantly higher, indicating a significant increase of grain numbers and thereby yield production. Testing another cereal plant, Ninkovic et al. exposed barley plants to methyl salicylate (MeSA) and then investigated its biological effects on the bird cherry-oat aphid *Rhopalosiphum padi* after different time intervals. They found that aphid settlement and behavior were negatively affected on MeSA-exposed plants due to subsequent metabolic changes in the released volatiles and phloem content.

In conclusion, this Research Topic identifies possible reasons why practical application of defense inducers, on farms for crop protection, is still low and highlights that more research under field conditions is still needed in future to further develop defense inducers. This would facilitate their adoption in IPM programs as environmentally safe and IPM-compatible agrochemicals to manage insect pests. The demand for alternatives is increasing due to restrictions in availability of conventional pesticides. To this end, the effects of defense inducers on (i) plant growth, productivity and yield, (ii) multiple plant antagonists, and (iii) effectiveness across a range of crop germplasm, still needs to be explored.

## Author Contributions

IS, YL, and TB contributed to organizing this Research Topic. IS wrote the first draft of this editorial based on the contributed articles. All authors contributed to the article and approved the submitted version.

## Conflict of Interest

The authors declare that the research was conducted in the absence of any commercial or financial relationships that could be construed as a potential conflict of interest.

## Publisher's Note

All claims expressed in this article are solely those of the authors and do not necessarily represent those of their affiliated organizations, or those of the publisher, the editors and the reviewers. Any product that may be evaluated in this article, or claim that may be made by its manufacturer, is not guaranteed or endorsed by the publisher.
